# 4-(*sec*-Butyl­amino)-3-nitro­benzoic acid

**DOI:** 10.1107/S1600536809021655

**Published:** 2009-06-13

**Authors:** Shivanagere Nagojappa Narendra Babu, Aisyah Saad Abdul Rahim, Shafida Abd Hamid, Chin Sing Yeap, Hoong-Kun Fun

**Affiliations:** a School of Pharmaceutical Sciences, Universiti Sains Malaysia, 11800 USM, Penang, Malaysia; b Kulliyyah of Science, International Islamic University Malaysia (IIUM), Jalan Istana, Bandar Indera Mahkota, 25200, Kuantan, Pahang, Malaysia; c X-ray Crystallography Unit, School of Physics, Universiti Sains Malaysia, 11800 USM, Penang, Malaysia

## Abstract

The asymmetric unit of title compound, C_11_H_14_N_2_O_4_, consists of two crystallographically independent mol­ecules (*A* and *B*). In each, intra­molecular N—H⋯O hydrogen bonds generate *S*(6) ring motifs. The mean plane of the nitro group forms dihedral angles of 4.5 (3) and 0.5 (3)° with the benzene ring in mol­ecules *A* and *B*, respectively. In mol­ecule *A*, there is disorder of the butyl­amino group which corresponds to an approximate 180° rotation about the N—C(H) bond, forming two sites with refined occupancies of 0.722 (6) and 0.278 (6). Mol­ecule *B* is similarly disordered but in addition there is further rotational disorder about the C(H)—C(H_2_) bond giving a ratio of occupancies for three components of 0.42:0.35:0.23. In the crystal structure, inter­molecular O—H⋯O hydrogen bonds link mol­ecules into centrosymmetric dimers generating *R*_2_^2^(8) ring motifs. The crystal structure is also stabilized by weak inter­molecular C—H⋯O inter­actions.

## Related literature

For the synthesis of bioactive heterocycles using nitro benzoic acid derivatives as the starting materials, see: Burgey *et al.* (2006 ▶); Ishida *et al.* (2006 ▶); Semple *et al.* (2006 ▶); Narendra Babu *et al.* (2009 ▶). For hydrogen-bond graph-set motifs, see: Bernstein *et al.* (1995 ▶). For the stability of the temperature controller used for the data collection, see: Cosier & Glazer (1986 ▶).
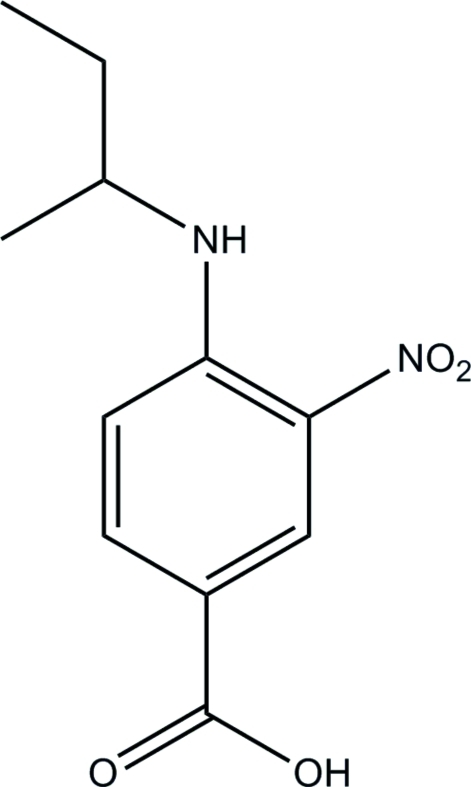


## Experimental

### 

#### Crystal data

C_11_H_14_N_2_O_4_
*M* *_r_* = 238.24
Monoclinic, 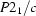

*a* = 6.9722 (4) Å
*b* = 15.7250 (8) Å
*c* = 21.8111 (11) Å
β = 101.896 (3)°
*V* = 2340.0 (2) Å^3^
*Z* = 8
Mo *K*α radiation
μ = 0.10 mm^−1^
*T* = 110 K
0.57 × 0.08 × 0.04 mm

#### Data collection

Bruker SMART APEXII CCD area-detector diffractometer
Absorption correction: multi-scan (**SADABS**; Bruker, 2005 ▶) *T*_min_ = 0.922, *T*_max_ = 0.995
23247 measured reflections
4600 independent reflections
3357 reflections with *I* > 2σ(*I*)
*R*_int_ = 0.049

#### Refinement

*R*[*F* ^2^ > 2σ(*F* ^2^)] = 0.070
*wR*(*F* ^2^) = 0.165
*S* = 1.08
4600 reflections
335 parameters
2 restraints
H atoms treated by a mixture of independent and constrained refinement
Δρ_max_ = 0.56 e Å^−3^
Δρ_min_ = −0.60 e Å^−3^

### 

Data collection: *APEX2* (Bruker, 2005 ▶); cell refinement: *SAINT* (Bruker, 2005 ▶); data reduction: *SAINT*; program(s) used to solve structure: *SHELXTL* (Sheldrick, 2008 ▶); program(s) used to refine structure: *SHELXTL*; molecular graphics: *SHELXTL*; software used to prepare material for publication: *SHELXTL* and *PLATON* (Spek, 2009 ▶).

## Supplementary Material

Crystal structure: contains datablocks global, I. DOI: 10.1107/S1600536809021655/lh2837sup1.cif

Structure factors: contains datablocks I. DOI: 10.1107/S1600536809021655/lh2837Isup2.hkl

Additional supplementary materials:  crystallographic information; 3D view; checkCIF report

## Figures and Tables

**Table 1:** Hydrogen-bond geometry (Å, °)

*D*—H⋯*A*	*D*—H	H⋯*A*	*D*⋯*A*	*D*—H⋯*A*
O1*A*—H1*OA*⋯O2*A*^i^	0.82	1.83	2.646 (3)	175
O1*B*—H1*OB*⋯O2*B*^ii^	0.82	1.80	2.618 (3)	172
N2*A*—H2*NA*⋯O4*A*	0.80 (3)	1.97 (3)	2.630 (4)	139 (3)
N2*B*—H2*NB*⋯O4*B*	0.88 (3)	1.90 (3)	2.627 (4)	139 (3)
C5*A*—H5*AA*⋯O3*A*^iii^	0.93	2.44	3.316 (4)	157
C5*B*—H5*BA*⋯O3*B*^iv^	0.93	2.47	3.253 (4)	142

Symmetry codes: (i) 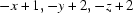; (ii) 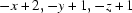; (iii) 
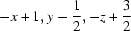
; (iv) 
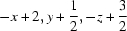
.

**Fig. 1.**
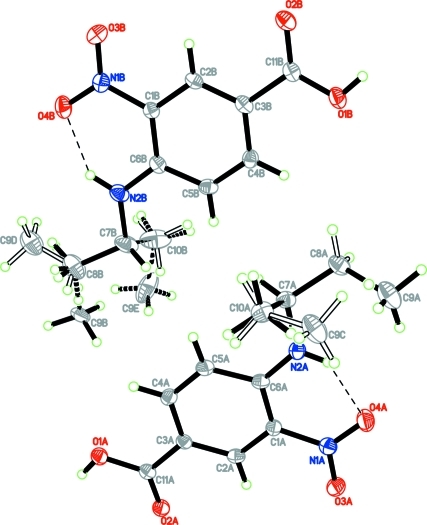
The molecular structure of the title compound with atom labels and 30% probability displacement ellipsoids for non-H atoms. Intramolecular hydrogen bonds are shown as dashed lines. All disorder components are shown.

**Fig. 2.**
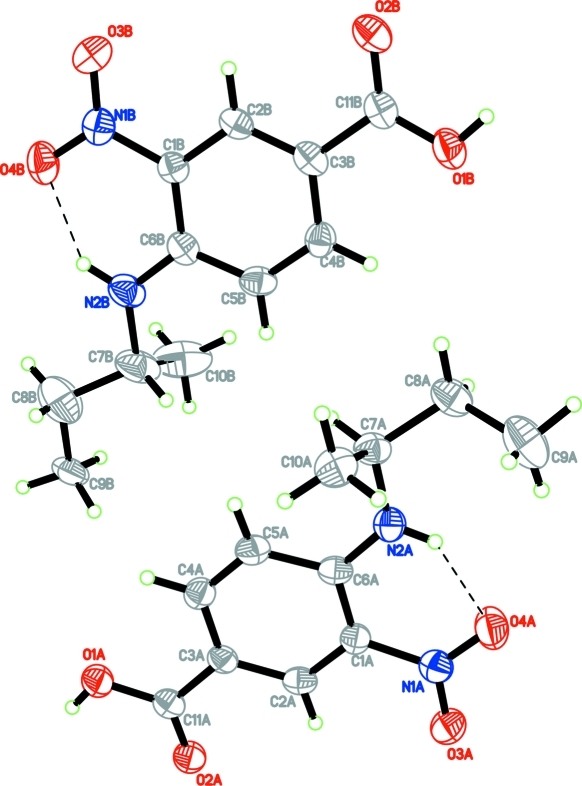
The major disorder component of the title compound with atom labels and 50% probability displacement ellipsoids for non-H atoms. Intramolecular hydrogen bonds are shown as dashed lines.

**Fig. 3.**
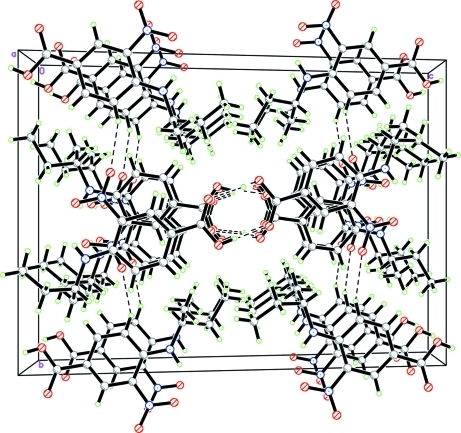
The crystal packing of the title compound, showing the centrosymmetric dimers. Intermolecular hydrogen bonds are shown as dashed lines. Only the major disorder component is shown.

## supplementary crystallographic information

### Comment

The synthesis of bioactive heterocycles such as substituted benzimidazolones, benzimidazoles and 1-substituted benzotriazole carboxylic acids, (Burgey *et al.*, 2006; Ishida *et al.*, 2006; Semple *et al.*, 2006), can be achieved from nitro benzoic acid derivatives as the starting materials. As part of our ongoing synthesis programme, (Narendra Babu *et al.*, 2009), we have synthesized the title compound as an intermediate and herein present its crystal structure.

The asymmetric unit of title compound (I), (Figs. 1 and 2), consists of two crystallographically independent molecule A and B. Intramolecular N2A—H2NA···O4A and N2B—H2NB···O4B hydrogen bonds generate *S*(6) ring motifs (Bernstein *et al.*, 1995). The nitro group for molecule A is almost planar with the benzene ring whereas the nitro group for molecule B is essentially coplanar with benzene ring [dihedral angle, A = 4.5 (3)° and B = 0.5 (3)°].

In molecule A there is disorder of the butylamino group which corresponds to an approximate 180° rotation about the N-C(H) bond forming two sites with refined occupancies of 0.722 (6) and 0.278 (6). Molecule B is similarly disordered but in addition there is further rotational disorder about the C(H)-C(H_2_) bond giving a ratio of occupancies for three components of 0.42:0.35:0.23 (see Fig. 1).

In the crystal structure, intermolecular O—H···O hydrogen bonds (see Table 1) link molecules into centrosymmetric dimers (Fig. 3) to generate *R*_2_^2^(8) ring motifs (Bernstein *et al.*, 1995). The crystal structure is further stabilized by weak intermolecular C—H···O interactions (Table 1).

### Experimental

Ethyl 4-(*sec*-butylamino)-3-nitro-benzoate (0.2 g, 0.00075 mol) and KOH (0.084 g, 0.0015 mol) was refluxed in aqueous ethanol (5 ml) for 3 h. After completion of the reaction, ethanol was distilled off and the reaction mixture was diluted with water (5 ml). The aqueous layer was washed with dichloromethane (5 ml x 2) and acidified with concentrated hydrochloric acid to afford yellow solid. Recrystallisation of the crude product with hot ethyl acetate afforded yellow crystals.

### Refinement

Atoms H2NA and H2NB were located in a difference Fourier map and refined freely. The H-atoms of the hydroxy groups were positioned using a rotating group model and constrained with a fixed distance of 0.82 Å. The H-atoms for C8A, C10A, C8B and C10B were positioned geometrically and refined as riding with the parent atom with U_iso_(H) = 1.2 and 1.5 U_eq_(C). The rest of the hydrogen atoms were positioned geometrically and refined using a riding model with C-H = 0.93–0.96 Å and U_iso_(H) = 1.2 and 1.5 U_eq_(C). A rotating-group model was applied for the methyl groups. The methyl group of molecule A is disordered over two positions with refined site-occupancy ratio of 0.722 (6) : 0.278 (6), whereas the methyl group of molecule B is treated as disordered over three positions with refined site-occupancy ratio of 0.413 (9) : 0.327 (8) : 0.237 (9) with SUMP command equal to 1.0 (1). For the final refinement, the site-occupancy ratio of molecule B is fixed to 0.42 : 0.35 : 0.23. The same U^ij^ parameters were used for atom pairs C9D/C9A, C9A/C8B and C9A/C9C. The C9E–C10B bond was refined with C-C distance restraint of 1.40 Å.

### Crystal data

**Table:**

C_11_H_14_N_2_O_4_	*F*(000) = 1008
*M**_r_* = 238.24	*D*_x_ = 1.353 Mg m^−^^3^
Monoclinic, *P*2_1_/*c*	Mo *K*α radiation, λ = 0.71073 Å
Hall symbol: -P 2ybc	Cell parameters from 5403 reflections
*a* = 6.9722 (4) Å	θ = 2.3–26.2°
*b* = 15.7250 (8) Å	µ = 0.10 mm^−^^1^
*c* = 21.8111 (11) Å	*T* = 110 K
β = 101.896 (3)°	Needle, yellow
*V* = 2340.0 (2) Å^3^	0.57 × 0.08 × 0.04 mm
*Z* = 8	

### Data collection

**Table:**

Bruker SMART APEXII CCD area-detector diffractometer	4600 independent reflections
Radiation source: fine-focus sealed tube	3357 reflections with *I* > 2σ(*I*)
graphite	*R*_int_ = 0.049
φ and ω scans	θ_max_ = 26.0°, θ_min_ = 1.9°
Absorption correction: multi-scan (*SADABS*; Bruker, 2005)	*h* = −8→8
*T*_min_ = 0.922, *T*_max_ = 0.995	*k* = −16→19
23247 measured reflections	*l* = −26→26

### Refinement

**Table:**

Refinement on *F*^2^	Primary atom site location: structure-invariant direct methods
Least-squares matrix: full	Secondary atom site location: difference Fourier map
*R*[*F*^2^ > 2σ(*F*^2^)] = 0.070	Hydrogen site location: inferred from neighbouring sites
*wR*(*F*^2^) = 0.165	H atoms treated by a mixture of independent and constrained refinement
*S* = 1.08	*w* = 1/[σ^2^(*F*_o_^2^) + (0.0456*P*)^2^ + 3.6565*P*] where *P* = (*F*_o_^2^ + 2*F*_c_^2^)/3
4600 reflections	(Δ/σ)_max_ < 0.001
335 parameters	Δρ_max_ = 0.56 e Å^−^^3^
2 restraints	Δρ_min_ = −0.59 e Å^−^^3^

### Special details

**Table:**

Experimental. The crystal was placed in the cold stream of an Oxford Cyrosystems Cobra open-flow nitrogen cryostat (Cosier & Glazer, 1986) operating at 110.0 (1)K.
Geometry. All esds (except the esd in the dihedral angle between two l.s. planes) are estimated using the full covariance matrix. The cell esds are taken into account individually in the estimation of esds in distances, angles and torsion angles; correlations between esds in cell parameters are only used when they are defined by crystal symmetry. An approximate (isotropic) treatment of cell esds is used for estimating esds involving l.s. planes.
Refinement. Refinement of F^2^ against ALL reflections. The weighted R-factor wR and goodness of fit S are based on F^2^, conventional R-factors R are based on F, with F set to zero for negative F^2^. The threshold expression of F^2^ > 2sigma(F^2^) is used only for calculating R-factors(gt) etc. and is not relevant to the choice of reflections for refinement. R-factors based on F^2^ are statistically about twice as large as those based on F, and R- factors based on ALL data will be even larger.

### Fractional atomic coordinates and isotropic or equivalent isotropic displacement parameters (Å^2^)

**Table:**

	*x*	*y*	*z*	*U*_iso_*/*U*_eq_	Occ. (<1)
O1A	0.4975 (3)	0.90262 (13)	0.95499 (9)	0.0352 (5)	
H1OA	0.4983	0.9220	0.9899	0.053*	
O2A	0.5198 (3)	1.04071 (13)	0.93205 (9)	0.0347 (5)	
O3A	0.5205 (3)	1.11848 (13)	0.71999 (10)	0.0404 (6)	
O4A	0.5426 (4)	1.02816 (15)	0.64713 (10)	0.0477 (6)	
N1A	0.5280 (4)	1.04472 (16)	0.70177 (12)	0.0325 (6)	
N2A	0.5194 (4)	0.86394 (17)	0.66720 (12)	0.0310 (6)	
C1A	0.5195 (4)	0.97566 (18)	0.74513 (13)	0.0272 (6)	
C2A	0.5183 (4)	0.99913 (19)	0.80664 (13)	0.0276 (6)	
H2AA	0.5215	1.0564	0.8174	0.033*	
C3A	0.5126 (4)	0.93828 (18)	0.85192 (13)	0.0260 (6)	
C4A	0.5080 (4)	0.85228 (19)	0.83378 (14)	0.0304 (7)	
H4AA	0.5045	0.8104	0.8636	0.036*	
C5A	0.5086 (4)	0.82863 (19)	0.77366 (13)	0.0310 (7)	
H5AA	0.5044	0.7711	0.7636	0.037*	
C6A	0.5155 (4)	0.88925 (18)	0.72596 (13)	0.0271 (6)	
C7A	0.5153 (4)	0.77568 (19)	0.64471 (14)	0.0324 (7)	
H7AA	0.5997	0.7415	0.6770	0.039*	
C8A	0.6020 (5)	0.7739 (2)	0.58601 (14)	0.0415 (8)	
H8AA	0.6047	0.7163	0.5716	0.050*	0.722 (6)
H8AB	0.7345	0.7944	0.5961	0.050*	0.722 (6)
H8AC	0.6047	0.7163	0.5716	0.062*	0.278 (6)
H8AD	0.7345	0.7944	0.5961	0.062*	0.278 (6)
H8AE	0.5210	0.8065	0.5534	0.062*	0.278 (6)
C9A	0.4827 (11)	0.8285 (4)	0.5329 (3)	0.0793 (11)	0.722 (6)
H9AA	0.5300	0.8193	0.4951	0.119*	0.722 (6)
H9AB	0.4967	0.8875	0.5443	0.119*	0.722 (6)
H9AC	0.3469	0.8127	0.5262	0.119*	0.722 (6)
C9C	0.186 (3)	0.7705 (12)	0.5685 (6)	0.0793 (11)	0.278 (6)
H9CA	0.0556	0.7473	0.5620	0.119*	0.278 (6)
H9CB	0.2478	0.7522	0.5354	0.119*	0.278 (6)
H9CC	0.1786	0.8315	0.5686	0.119*	0.278 (6)
C10A	0.3092 (5)	0.7385 (2)	0.63375 (15)	0.0407 (8)	
H10A	0.2611	0.7413	0.6719	0.061*	0.722 (6)
H10B	0.2244	0.7706	0.6018	0.061*	0.722 (6)
H10C	0.3120	0.6803	0.6207	0.061*	0.722 (6)
H10D	0.2465	0.7554	0.6671	0.049*	0.278 (6)
H10E	0.3156	0.6775	0.6334	0.049*	0.278 (6)
C11A	0.5106 (4)	0.96526 (19)	0.91601 (13)	0.0279 (6)	
O1B	0.9570 (3)	0.58178 (15)	0.55036 (10)	0.0404 (6)	
H1OB	0.9671	0.5690	0.5147	0.061*	
O2B	1.0142 (3)	0.44245 (15)	0.56615 (10)	0.0401 (6)	
O3B	1.0282 (3)	0.32730 (14)	0.77010 (10)	0.0411 (6)	
O4B	0.9980 (3)	0.40380 (15)	0.84984 (10)	0.0446 (6)	
N1B	1.0041 (3)	0.39668 (17)	0.79356 (12)	0.0330 (6)	
N2B	0.9466 (4)	0.5691 (2)	0.83945 (12)	0.0393 (7)	
C1B	0.9824 (4)	0.47178 (18)	0.75471 (13)	0.0268 (6)	
C2B	0.9906 (4)	0.46020 (19)	0.69198 (13)	0.0268 (6)	
H2BA	1.0104	0.4060	0.6773	0.032*	
C3B	0.9699 (4)	0.5281 (2)	0.65165 (13)	0.0289 (7)	
C4B	0.9402 (4)	0.60957 (19)	0.67420 (13)	0.0308 (7)	
H4BA	0.9256	0.6558	0.6471	0.037*	
C5B	0.9326 (4)	0.6215 (2)	0.73584 (14)	0.0333 (7)	
H5BA	0.9118	0.6761	0.7495	0.040*	
C6B	0.9549 (4)	0.5542 (2)	0.77919 (13)	0.0298 (7)	
C7B	0.9414 (5)	0.6537 (2)	0.86791 (16)	0.0504 (10)	
H7BA	0.8414	0.6878	0.8405	0.061*	
C8B	0.8803 (8)	0.6422 (3)	0.92986 (19)	0.0793 (11)	
H8BA	0.9565	0.5983	0.9541	0.095*	0.42
H8BB	0.7449	0.6257	0.9225	0.095*	0.42
H8BC	0.7414	0.6519	0.9212	0.095*	0.35
H8BD	0.9383	0.6887	0.9556	0.095*	0.35
H8BE	0.7565	0.6132	0.9231	0.119*	0.23
H8BF	0.9767	0.6095	0.9580	0.119*	0.23
H8BG	0.8675	0.6972	0.9477	0.119*	0.23
C9B	0.9071 (12)	0.7303 (5)	0.9672 (4)	0.044 (2)	0.42
H9BA	0.8896	0.7213	1.0092	0.066*	0.42
H9BB	0.8114	0.7704	0.9465	0.066*	0.42
H9BC	1.0363	0.7522	0.9682	0.066*	0.42
C9D	0.914 (2)	0.5772 (9)	0.9672 (5)	0.0793 (11)	0.35
H9DA	0.8517	0.5277	0.9461	0.119*	0.35
H9DB	0.8613	0.5881	1.0038	0.119*	0.35
H9DC	1.0523	0.5678	0.9792	0.119*	0.35
C9E	1.092 (3)	0.7665 (8)	0.9122 (8)	0.081 (7)	0.23
H9EA	1.1756	0.8140	0.9085	0.121*	0.23
H9EB	1.1137	0.7487	0.9552	0.121*	0.23
H9EC	0.9577	0.7829	0.8983	0.121*	0.23
C10B	1.1359 (6)	0.6989 (3)	0.8750 (2)	0.0694 (13)	
H10F	1.1684	0.7036	0.8345	0.104*	0.42
H10G	1.1268	0.7547	0.8920	0.104*	0.42
H10H	1.2360	0.6671	0.9024	0.104*	0.42
H10I	1.1684	0.7036	0.8345	0.104*	0.35
H10J	1.1268	0.7547	0.8920	0.104*	0.35
H10K	1.2360	0.6671	0.9024	0.104*	0.35
H10L	1.1610	0.7152	0.8350	0.083*	0.23
H10M	1.2428	0.6652	0.8971	0.083*	0.23
C11B	0.9816 (4)	0.5153 (2)	0.58518 (13)	0.0307 (7)	
H2NA	0.523 (5)	0.903 (2)	0.6440 (15)	0.031 (9)*	
H2NB	0.961 (5)	0.522 (2)	0.8616 (17)	0.053 (12)*	

### Atomic displacement parameters (Å^2^)

**Table:**

	*U*^11^	*U*^22^	*U*^33^	*U*^12^	*U*^13^	*U*^23^
O1A	0.0455 (13)	0.0321 (12)	0.0299 (11)	−0.0005 (10)	0.0117 (10)	−0.0033 (9)
O2A	0.0423 (13)	0.0312 (12)	0.0315 (11)	−0.0004 (10)	0.0095 (9)	−0.0036 (9)
O3A	0.0496 (14)	0.0281 (12)	0.0448 (13)	−0.0035 (10)	0.0130 (11)	0.0000 (10)
O4A	0.0692 (17)	0.0418 (14)	0.0355 (13)	−0.0061 (12)	0.0187 (12)	−0.0002 (10)
N1A	0.0310 (14)	0.0316 (15)	0.0359 (15)	−0.0030 (11)	0.0088 (11)	−0.0023 (11)
N2A	0.0318 (14)	0.0326 (15)	0.0279 (14)	0.0001 (11)	0.0048 (11)	0.0012 (12)
C1A	0.0183 (13)	0.0321 (16)	0.0307 (15)	0.0016 (12)	0.0037 (11)	0.0047 (12)
C2A	0.0180 (13)	0.0289 (15)	0.0349 (16)	0.0000 (11)	0.0033 (12)	−0.0042 (13)
C3A	0.0195 (13)	0.0302 (16)	0.0287 (15)	0.0015 (11)	0.0056 (12)	−0.0003 (12)
C4A	0.0260 (15)	0.0306 (16)	0.0344 (16)	0.0010 (12)	0.0058 (13)	0.0037 (13)
C5A	0.0329 (16)	0.0271 (16)	0.0325 (16)	0.0023 (13)	0.0052 (13)	−0.0033 (13)
C6A	0.0185 (13)	0.0298 (16)	0.0318 (15)	0.0020 (12)	0.0024 (12)	−0.0032 (13)
C7A	0.0341 (16)	0.0321 (17)	0.0294 (15)	0.0024 (13)	0.0027 (13)	−0.0061 (13)
C8A	0.0405 (18)	0.050 (2)	0.0341 (17)	−0.0005 (16)	0.0078 (14)	−0.0102 (15)
C9A	0.112 (3)	0.091 (3)	0.0410 (17)	0.009 (2)	0.0301 (19)	−0.0072 (17)
C9C	0.112 (3)	0.091 (3)	0.0410 (17)	0.009 (2)	0.0301 (19)	−0.0072 (17)
C10A	0.0396 (18)	0.0367 (18)	0.0433 (19)	−0.0024 (15)	0.0032 (15)	−0.0039 (15)
C11A	0.0189 (14)	0.0338 (17)	0.0301 (16)	−0.0009 (12)	0.0032 (12)	−0.0022 (13)
O1B	0.0443 (13)	0.0517 (14)	0.0261 (11)	0.0019 (11)	0.0093 (10)	0.0018 (10)
O2B	0.0418 (13)	0.0476 (14)	0.0312 (12)	−0.0007 (11)	0.0078 (10)	−0.0070 (10)
O3B	0.0414 (13)	0.0321 (13)	0.0478 (14)	0.0005 (10)	0.0043 (11)	0.0024 (11)
O4B	0.0488 (14)	0.0551 (15)	0.0301 (12)	0.0012 (12)	0.0087 (10)	0.0109 (11)
N1B	0.0239 (13)	0.0383 (16)	0.0358 (14)	−0.0015 (11)	0.0041 (11)	0.0050 (12)
N2B	0.0423 (16)	0.0478 (18)	0.0271 (14)	0.0093 (13)	0.0054 (12)	−0.0035 (13)
C1B	0.0183 (13)	0.0331 (16)	0.0287 (15)	0.0008 (12)	0.0042 (11)	0.0007 (12)
C2B	0.0164 (13)	0.0342 (16)	0.0299 (15)	−0.0011 (12)	0.0048 (11)	−0.0045 (13)
C3B	0.0163 (13)	0.0415 (18)	0.0279 (15)	−0.0023 (12)	0.0026 (11)	−0.0041 (13)
C4B	0.0279 (15)	0.0340 (17)	0.0297 (15)	−0.0002 (13)	0.0039 (12)	0.0048 (13)
C5B	0.0280 (15)	0.0353 (17)	0.0354 (17)	0.0059 (13)	0.0035 (13)	−0.0045 (14)
C6B	0.0215 (14)	0.0421 (18)	0.0247 (15)	0.0039 (13)	0.0020 (12)	0.0011 (13)
C7B	0.054 (2)	0.061 (2)	0.0341 (18)	0.0158 (19)	0.0043 (16)	−0.0147 (17)
C8B	0.112 (3)	0.091 (3)	0.0410 (17)	0.009 (2)	0.0301 (19)	−0.0072 (17)
C9B	0.047 (5)	0.047 (5)	0.042 (4)	0.010 (4)	0.020 (4)	−0.008 (4)
C9D	0.112 (3)	0.091 (3)	0.0410 (17)	0.009 (2)	0.0301 (19)	−0.0072 (17)
C9E	0.113 (17)	0.041 (10)	0.080 (14)	−0.033 (11)	0.000 (12)	0.013 (10)
C10B	0.059 (3)	0.065 (3)	0.071 (3)	0.012 (2)	−0.018 (2)	−0.036 (2)
C11B	0.0210 (14)	0.0426 (19)	0.0275 (15)	−0.0022 (13)	0.0031 (12)	−0.0003 (14)

### Geometric parameters (Å, °)

**Table:**

O1A—C11A	1.317 (3)	N2B—H2NB	0.88 (4)
O1A—H1OA	0.8200	C1B—C2B	1.393 (4)
O2A—C11A	1.235 (3)	C1B—C6B	1.429 (4)
O3A—N1A	1.231 (3)	C2B—C3B	1.372 (4)
O4A—N1A	1.244 (3)	C2B—H2BA	0.9300
N1A—C1A	1.449 (4)	C3B—C4B	1.403 (4)
N2A—C6A	1.348 (4)	C3B—C11B	1.483 (4)
N2A—C7A	1.470 (4)	C4B—C5B	1.369 (4)
N2A—H2NA	0.80 (3)	C4B—H4BA	0.9300
C1A—C2A	1.393 (4)	C5B—C6B	1.406 (4)
C1A—C6A	1.420 (4)	C5B—H5BA	0.9300
C2A—C3A	1.382 (4)	C7B—C8B	1.509 (5)
C2A—H2AA	0.9300	C7B—C10B	1.511 (6)
C3A—C4A	1.408 (4)	C7B—H7BA	0.9800
C3A—C11A	1.464 (4)	C8B—C9D	1.297 (14)
C4A—C5A	1.364 (4)	C8B—C9B	1.599 (9)
C4A—H4AA	0.9300	C8B—H8BA	0.9601
C5A—C6A	1.419 (4)	C8B—H8BB	0.9600
C5A—H5AA	0.9300	C8B—H8BC	0.9600
C7A—C10A	1.524 (4)	C8B—H8BD	0.9600
C7A—C8A	1.525 (4)	C8B—H8BE	0.9600
C7A—H7AA	0.9800	C8B—H8BF	0.9601
C8A—C9A	1.541 (7)	C8B—H8BG	0.9600
C8A—H8AA	0.9601	C9B—H8BD	0.7494
C8A—H8AB	0.9599	C9B—H8BG	0.6932
C8A—H8AC	0.9601	C9B—H9BA	0.9600
C8A—H8AD	0.9599	C9B—H9BB	0.9600
C8A—H8AE	0.9600	C9B—H9BC	0.9600
C9A—H8AE	0.5846	C9D—H8BA	0.5627
C9A—H9AA	0.9600	C9D—H8BB	1.5624
C9A—H9AB	0.9600	C9D—H8BE	1.4173
C9A—H9AC	0.9600	C9D—H8BF	0.7266
C9C—C10A	1.586 (16)	C9D—H9DA	0.9600
C9C—H9CA	0.9600	C9D—H9DB	0.9600
C9C—H9CB	0.9600	C9D—H9DC	0.9600
C9C—H9CC	0.9600	C9E—C10B	1.408 (9)
C9C—H10B	0.7203	C9E—H9EA	0.9600
C10A—H10A	0.9601	C9E—H9EB	0.9600
C10A—H10B	0.9599	C9E—H9EC	0.9600
C10A—H10C	0.9600	C9E—H10G	0.5739
C10A—H10D	0.9601	C9E—H10J	0.5739
C10A—H10E	0.9600	C10B—H10F	0.9600
O1B—C11B	1.283 (4)	C10B—H10G	0.9599
O1B—H1OB	0.8200	C10B—H10H	0.9599
O2B—C11B	1.254 (4)	C10B—H10I	0.9600
O3B—N1B	1.231 (3)	C10B—H10J	0.9599
O4B—N1B	1.242 (3)	C10B—H10K	0.9599
N1B—C1B	1.443 (4)	C10B—H10L	0.9600
N2B—C6B	1.348 (4)	C10B—H10M	0.9601
N2B—C7B	1.472 (4)		
			
C11A—O1A—H1OA	109.5	C5B—C6B—C1B	115.8 (3)
O3A—N1A—O4A	121.6 (3)	N2B—C7B—C8B	107.8 (3)
O3A—N1A—C1A	119.0 (2)	N2B—C7B—C10B	111.7 (3)
O4A—N1A—C1A	119.4 (2)	C8B—C7B—C10B	112.2 (3)
C6A—N2A—C7A	126.4 (3)	N2B—C7B—H7BA	108.4
C6A—N2A—H2NA	113 (2)	C8B—C7B—H7BA	108.4
C7A—N2A—H2NA	121 (2)	C10B—C7B—H7BA	108.4
C2A—C1A—C6A	122.2 (3)	C9D—C8B—C7B	127.5 (7)
C2A—C1A—N1A	116.0 (3)	C9D—C8B—C9B	112.1 (7)
C6A—C1A—N1A	121.7 (3)	C7B—C8B—C9B	109.1 (5)
C3A—C2A—C1A	120.8 (3)	C7B—C8B—H8BA	110.6
C3A—C2A—H2AA	119.6	C9B—C8B—H8BA	110.4
C1A—C2A—H2AA	119.6	C9D—C8B—H8BB	86.3
C2A—C3A—C4A	117.8 (3)	C7B—C8B—H8BB	109.4
C2A—C3A—C11A	119.3 (3)	C9B—C8B—H8BB	109.1
C4A—C3A—C11A	122.9 (3)	H8BA—C8B—H8BB	108.2
C5A—C4A—C3A	121.9 (3)	C9D—C8B—H8BC	107.0
C5A—C4A—H4AA	119.1	C7B—C8B—H8BC	105.3
C3A—C4A—H4AA	119.1	C9B—C8B—H8BC	88.3
C4A—C5A—C6A	121.9 (3)	H8BA—C8B—H8BC	130.2
C4A—C5A—H5AA	119.0	C9D—C8B—H8BD	103.5
C6A—C5A—H5AA	119.0	C7B—C8B—H8BD	105.7
N2A—C6A—C5A	120.6 (3)	H8BA—C8B—H8BD	96.2
N2A—C6A—C1A	124.0 (3)	H8BB—C8B—H8BD	125.8
C5A—C6A—C1A	115.4 (3)	H8BC—C8B—H8BD	106.3
N2A—C7A—C10A	111.5 (2)	C9D—C8B—H8BE	76.2
N2A—C7A—C8A	108.3 (3)	C7B—C8B—H8BE	109.3
C10A—C7A—C8A	112.7 (3)	C9B—C8B—H8BE	119.7
N2A—C7A—H7AA	108.1	H8BA—C8B—H8BE	97.0
C10A—C7A—H7AA	108.1	H8BD—C8B—H8BE	135.1
C8A—C7A—H7AA	108.1	C7B—C8B—H8BF	110.3
C7A—C8A—C9A	112.0 (3)	C9B—C8B—H8BF	98.3
C7A—C8A—H8AA	109.2	H8BB—C8B—H8BF	119.8
C9A—C8A—H8AA	108.9	H8BC—C8B—H8BF	139.1
C7A—C8A—H8AB	109.3	H8BD—C8B—H8BF	83.0
C9A—C8A—H8AB	109.3	H8BE—C8B—H8BF	109.5
H8AA—C8A—H8AB	108.0	C9D—C8B—H8BG	118.4
C7A—C8A—H8AC	109.2	C7B—C8B—H8BG	108.8
C9A—C8A—H8AC	108.9	H8BA—C8B—H8BG	120.8
H8AB—C8A—H8AC	108.0	H8BB—C8B—H8BG	98.1
C7A—C8A—H8AD	109.3	H8BC—C8B—H8BG	76.2
C9A—C8A—H8AD	109.3	H8BE—C8B—H8BG	109.5
H8AA—C8A—H8AD	108.0	H8BF—C8B—H8BG	109.5
H8AC—C8A—H8AD	108.0	C8B—C9B—H9BA	109.5
C7A—C8A—H8AE	109.7	H8BD—C9B—H9BA	107.2
H8AA—C8A—H8AE	108.1	C8B—C9B—H9BB	109.5
H8AB—C8A—H8AE	112.5	H8BD—C9B—H9BB	129.6
H8AC—C8A—H8AE	108.1	C8B—C9B—H9BC	109.5
H8AD—C8A—H8AE	112.5	H8BD—C9B—H9BC	89.1
C8A—C9A—H9AA	109.5	H8BA—C9D—H8BB	78.9
H8AE—C9A—H9AA	112.5	H8BA—C9D—H8BE	80.7
C8A—C9A—H9AB	109.5	C8B—C9D—H8BF	46.9
H8AE—C9A—H9AB	111.6	H8BB—C9D—H8BF	84.7
C8A—C9A—H9AC	109.5	H8BE—C9D—H8BF	87.8
H8AE—C9A—H9AC	104.2	C8B—C9D—H9DA	109.5
C10A—C9C—H9CA	109.5	C8B—C9D—H9DB	109.5
C10A—C9C—H9CB	109.5	H9DA—C9D—H9DB	109.5
H9CA—C9C—H9CB	109.5	C8B—C9D—H9DC	109.5
C10A—C9C—H9CC	109.5	H9DA—C9D—H9DC	109.5
H9CA—C9C—H9CC	109.5	H9DB—C9D—H9DC	109.5
H9CB—C9C—H9CC	109.5	C10B—C9E—H9EA	109.5
H9CA—C9C—H10B	107.3	C10B—C9E—H9EB	109.5
H9CB—C9C—H10B	128.6	C10B—C9E—H9EC	109.5
H9CC—C9C—H10B	90.2	H9EA—C9E—H10G	80.3
C7A—C10A—C9C	109.8 (7)	H9EB—C9E—H10G	130.4
C7A—C10A—H10A	109.5	H9EC—C9E—H10G	112.4
C9C—C10A—H10A	122.8	H9EA—C9E—H10J	80.3
C7A—C10A—H10B	109.3	H9EB—C9E—H10J	130.4
H10A—C10A—H10B	109.5	H9EC—C9E—H10J	112.4
C7A—C10A—H10C	109.6	C9E—C10B—C7B	96.8 (9)
C9C—C10A—H10C	94.4	C9E—C10B—H10F	126.4
H10A—C10A—H10C	109.5	C7B—C10B—H10F	108.6
H10B—C10A—H10C	109.5	C7B—C10B—H10G	109.9
C7A—C10A—H10D	109.7	H10F—C10B—H10G	109.5
C9C—C10A—H10D	110.1	C9E—C10B—H10H	104.5
H10B—C10A—H10D	95.6	C7B—C10B—H10H	109.9
H10C—C10A—H10D	122.0	H10F—C10B—H10H	109.5
C7A—C10A—H10E	109.9	H10G—C10B—H10H	109.5
C9C—C10A—H10E	109.1	C9E—C10B—H10I	126.4
H10A—C10A—H10E	94.5	C7B—C10B—H10I	108.6
H10B—C10A—H10E	122.9	H10G—C10B—H10I	109.5
H10D—C10A—H10E	108.2	H10H—C10B—H10I	109.5
O2A—C11A—O1A	122.8 (3)	C7B—C10B—H10J	109.9
O2A—C11A—C3A	122.6 (3)	H10F—C10B—H10J	109.5
O1A—C11A—C3A	114.5 (3)	H10H—C10B—H10J	109.5
C11B—O1B—H1OB	109.5	H10I—C10B—H10J	109.5
O3B—N1B—O4B	121.7 (3)	C9E—C10B—H10K	104.5
O3B—N1B—C1B	119.2 (2)	C7B—C10B—H10K	109.9
O4B—N1B—C1B	119.1 (3)	H10F—C10B—H10K	109.5
C6B—N2B—C7B	125.3 (3)	H10G—C10B—H10K	109.5
C6B—N2B—H2NB	111 (2)	H10I—C10B—H10K	109.5
C7B—N2B—H2NB	123 (2)	H10J—C10B—H10K	109.5
C2B—C1B—C6B	121.4 (3)	C9E—C10B—H10L	115.1
C2B—C1B—N1B	116.7 (3)	C7B—C10B—H10L	111.1
C6B—C1B—N1B	121.9 (3)	H10G—C10B—H10L	98.5
C3B—C2B—C1B	120.5 (3)	H10H—C10B—H10L	117.3
C3B—C2B—H2BA	119.7	H10J—C10B—H10L	98.5
C1B—C2B—H2BA	119.7	H10K—C10B—H10L	117.3
C2B—C3B—C4B	119.3 (3)	C9E—C10B—H10M	111.4
C2B—C3B—C11B	120.1 (3)	C7B—C10B—H10M	112.3
C4B—C3B—C11B	120.6 (3)	H10F—C10B—H10M	101.5
C5B—C4B—C3B	120.5 (3)	H10G—C10B—H10M	114.7
C5B—C4B—H4BA	119.7	H10I—C10B—H10M	101.5
C3B—C4B—H4BA	119.7	H10J—C10B—H10M	114.7
C4B—C5B—C6B	122.4 (3)	H10L—C10B—H10M	109.6
C4B—C5B—H5BA	118.8	O2B—C11B—O1B	124.0 (3)
C6B—C5B—H5BA	118.8	O2B—C11B—C3B	119.9 (3)
N2B—C6B—C5B	120.3 (3)	O1B—C11B—C3B	116.1 (3)
N2B—C6B—C1B	123.9 (3)		

### Hydrogen-bond geometry (Å, °)

**Table:**

*D*—H···*A*	*D*—H	H···*A*	*D*···*A*	*D*—H···*A*
O1A—H1OA···O2A^i^	0.82	1.83	2.646 (3)	175
O1B—H1OB···O2B^ii^	0.82	1.80	2.618 (3)	172
N2A—H2NA···O4A	0.80 (3)	1.97 (3)	2.630 (4)	139 (3)
N2B—H2NB···O4B	0.88 (3)	1.90 (3)	2.627 (4)	139 (3)
C5A—H5AA···O3A^iii^	0.93	2.44	3.316 (4)	157
C5B—H5BA···O3B^iv^	0.93	2.47	3.253 (4)	142

Symmetry codes: (i) −*x*+1, −*y*+2, −*z*+2; (ii) −*x*+2, −*y*+1, −*z*+1; (iii) −*x*+1, *y*−1/2, −*z*+3/2; (iv) −*x*+2, *y*+1/2, −*z*+3/2.
